# Comparison of Clinical Features and CT Temporal Changes Between Familial Clusters and Non-familial Patients With COVID-19 Pneumonia

**DOI:** 10.3389/fmed.2021.630802

**Published:** 2021-04-15

**Authors:** Shuyi Liu, Huanchu Yuan, Bin Zhang, Wei Li, Jingjing You, Jing Liu, Qingyang Zhong, Lu Zhang, Luyan Chen, Shaolin Li, Yujian Zou, Shuixing Zhang

**Affiliations:** ^1^Department of Radiology, The First Affiliated Hospital of Jinan University, Guangzhou, China; ^2^Department of Radiology, Dongguan People's Hospital, Dongguan, China; ^3^Guangdong Provincial Key Laboratory of Biomedical Imaging, Department of Radiology, The Fifth Affiliated Hospital of Sun Yat-sen University, Zhuhai, China; ^4^Dongguan Ninth People's Hospital, Dongguan, China

**Keywords:** coronavirus infections, pneumonia, tomography, X-ray computed, familial cluster

## Abstract

**Purpose:** This study aimed to compare the clinical characteristics, laboratory findings, and chest computed tomography (CT) findings of familial cluster (FC) and non-familial (NF) patients with coronavirus disease 2019 (COVID-19) pneumonia.

**Methods:** This retrospective study included 178 symptomatic adult patients with laboratory-confirmed COVID-19. The 178 patients were divided into FC (*n* = 108) and NF (*n* = 70) groups. Patients with at least two confirmed COVID-19 cases in their household were classified into the FC group. The clinical and laboratory features between the two groups were compared and so were the chest CT findings on-admission and end-hospitalization.

**Results:** Compared with the NF group, the FC group had a longer period of exposure (13.1 vs. 8.9 days, *p* < 0.001), viral shedding (21.5 vs. 15.9 days, *p* < 0.001), and hospital stay (39.2 vs. 22.2 days, *p* < 0.001). The FC group showed a higher number of involved lung lobes on admission (3.0 vs. 2.3, *p* = 0.017) and at end-hospitalization (3.6 vs. 1.7, *p* < 0.001) as well as higher sum severity CT scores at end-hospitalization (4.6 vs. 2.7, *p* = 0.005) than did the NF group. Conversely, the FC group had a lower lymphocyte count level (*p* < 0.001) and a significantly lower difference in the number of involved lung lobes (Δnumber) between admission and discharge (*p* < 0.001). Notably, more cases of severe or critical illness were observed in the FC group than in the NF group (*p* = 0.036).

**Conclusions:** Patients in the FC group had a worse clinical course and outcome than those in the NF group; thus, close monitoring during treatment and follow-ups after discharge would be beneficial for patients with familial infections.

## Introduction

In December 2019, the outbreak of coronavirus disease 2019 (COVID-19), which is caused by severe acute respiratory syndrome coronavirus 2 (SARS-CoV-2), spread rapidly, causing great concern worldwide. Jasper et al. (1) firstly reported a familial cluster of COVID-19, which indicated that this disease could be transmitted from person to person. Other related studies (2, 3) also confirmed this. It was observed that familial transmission enables SARS-CoV-2 to spread faster and for infections to become more widespread. It is considered that about 80% of the cluster transmission occurred in families in China (4), and transmission among family members likely remains an import route of transmission, especially in areas where families have many household members (5). In contrast, other coronavirus family members, such as the Middle East respiratory syndrome coronavirus (MERS-CoV) and severe acute respiratory syndrome coronavirus (SARS- CoV), cause diseases that mainly spread through non-socomial transmission. In fact, ~43.5–100% of these cases were related to transmission in hospitals, with MERS-CoV and SARS-CoV transmission within families accounting for only 13–21% and 22–39% of cases, respectively (6–8). The secondary attack rate in the familial transmission of COVID-19 has been described to range from 3 to 30% (5, 9). Furthermore, familial transmission is more critical for older individuals since they tend to stay at home, and the mortality rate for COVID-19 is markedly associated with age (10). Studies suggest that in the familial spread of COVID-19, adults are more likely to present with symptoms than are children (11).

The epidemiological, clinical, laboratory, and radiological findings of COVID-19 have been well-characterized (11–13). However, despite the ongoing spread of COVID-19, the differences between familial clusters and non-familial cases of COVID-19 are still not fully understood. Therefore, the aim of this study was to compare clinical characteristics, laboratory findings, and computed tomography (CT) findings between familial clusters and non-familial cases of COVID-19 pneumonia.

## Materials and Methods

This study was approved by the ethics committee; informed consent for this retrospective study was waived.

### Clinical Data Collection

A total of 178 symptomatic adult patients (≥18 years old) with COVID-19 pneumonia, laboratory-confirmed by the Centers for Disease Control and Prevention, were included in this retrospective cohort study (29 patients asymptomatic on admission were excluded; 16 patients were from familial clusters and 13 were not). Their data were collected from electronic medical records from January 23 to December 4, 2020. Individuals with a history of exposure were screened by the government. The subjects of surveillance included travelers from Hubei Province or other local areas affected by the pandemic and suspected individuals having close contacts with confirmed COVID-19 patients. They were tested for SARS-CoV-2 infection by reverse transcription-polymerase chain reaction (RT-PCR) tests using swab samples and quarantined at home or at a designated facility for 14 days. For some individuals, COVID-19 was detected through fever screening at local clinics. In the early stages of the pandemic, patients with positive RT-PCR results for SARS-CoV-2 would be admitted to the designated hospitals for treatment regardless of symptom severity. A diagnosis of COVID-19 and the severity of illness were determined according to the interim guidelines of the World Health Organization (14). The patients were admitted to three designated hospitals for COVID-19 treatment in Dongguan City and Zhuhai City of Guangdong Province, China: Dongguan People's Hospital, Dongguan Ninth People's Hospital, and the Fifth Affiliated Hospital of Sun Yat-Sen University.

The patients were divided into familial cluster (FC) and non-familial (NF) groups. Familial cluster infection referred to at least two confirmed COVID-19 cases found in a family. A common cause of SARS-CoV-2 infection is close contact or co-exposure between patients (15). In other words, patients with at least two confirmed cases of COVID-19 in their families were eligible for inclusions in the FC group. Patients in the NF group were totally isolated individuals for whom no secondary infection was observed within their families. We collected data on their initial clinical characteristics, laboratory findings upon admission, and clinical outcomes during hospitalization from electronic medical records. These data included age, sex, exposure history during the preceding 14 days, comorbidities, signs and symptoms, disease severity, number of intensive care unit (ICU) admissions, clinical outcomes, and length of different events. The duration of exposure to infection sources was defined as the duration of stay in the local areas affected by the pandemic or the duration of contact with COVID-19 patients. For the local residents of areas affected by the pandemic, the duration of exposure was defined as 14 days. The duration of viral shedding was defined as the number of days from the onset of symptoms to the first negative RT-PCR assay result followed by at least two subsequent negative RT-PCR results (16, 17). Biopsy specimens from COVID-19 patients were collected, and RT-PCR assays of nasal, throat, or rectal swabs were performed every 3–4 days during hospitalization: two consecutive negative RT-PCR test results with an interval of at least 1 day were required before patient discharge.

### CT Examinations

Chest CT scans were performed with three multi-detector CT scanners (GE Optima 520 Pro, America; Philips Brilliance iCT, Netherlands; KAIPU CT precision 32, China). Each patient was scanned from the lung apex to the diaphragm during a breath-hold at end full inspiration and at end normal-expiration. CT acquisition was executed as follows: (a) GE Optima 520 Pro and Philips Brilliance iCT, tube voltage, 120 kVp; tube current, 250 mA; slice thickness, 1.25 mm; slice spacing, 1.25 mm; (b) KAIPU CT precision 32, tube voltage, 120 kVp; tube current, automatic mA; slice thickness, 1.25 mm; slice spacing, 0.7 mm. No contrast agent was administered.

### CT Images Analysis

Chest CT was performed at two time points: on admission and at end-hospitalization. Patients for whom the time interval between symptom onset and admission CT examination was within 7 days were included in the initial CT analysis; those for whom the time interval between the last CT scan and discharge was within 3 days were included in the end-hospitalization CT analysis. Finally, 147 and 127 patients were included in the at admission and at end-hospitalization CT analyses, respectively. The differences in the sum severity CT scores (Δsum) and the number of involved lung lobes (Δnumber) between admission and end-hospitalization were calculated to determine the quantitative change in lung opacities over time. A total of 111 patients were eligible for the quantitative analysis of CT differences. CT images were independently assessed by two radiologists (with 22 and 23 years of experience in thoracic imaging), and discrepancies were resolved through discussion or by a third reviewer (with 25 years of experience in thoracic imaging). All CT images were reviewed using the lung algorithm (window width, 1,250 HU; window level, −600 HU) and mediastinal algorithm (window width, 350 HU; window level, 40 HU). The two radiologists classified the predominant patterns seen on chest CT as normal, ground-glass opacities (GGO, hazy areas of increased attenuation without obscuration of the underlying vessels); consolidation (homogeneous opacification of the parenchyma with obscuration of the underlying vessels); irregular linear opacities pattern; or mixed pattern (present with GGO, consolidation and irregular linear opacities). The distribution of lung abnormalities was recorded as either predominantly pleural (involving mainly the peripheral one-third of the lung), random (without predilection for pleural or central regions), diffuse (continuous involvement without respect to lung segments), or none (normal). Pleural effusion, lymphadenopathy (defined as a lymph node >1 cm in short-axis diameter), air bronchogram, enlarged pulmonary vessels, and pleural thickening were also recorded (18). Each of the five lung lobes was evaluated for the degree of involvement, which was classified as score 0 (0%), score 1 (1–25%), score 2 (26–50%), score 3 (51–75%), or score 4 (76–100%). The sum severity CT scores of the total lung were obtained by summing the scores of the five lobes with the maximum score of 20 (19).

### Statistical Analysis

Data were analyzed using SPSS Statistics version 24 (IBM Co., Armonk, NY, USA). Categorical variables were presented as counts (n) and percentages (%) and were compared between the groups using Chi-squared tests or Fisher's exact tests. Continuous variables were expressed as means and standard deviations or as medians and interquartile ranges and compared using two-sample independent *t*-tests. A *p* < 0.05 was considered to indicate statistical significance.

## Results

### Clinical Characteristics of Patients

Of the 178 symptomatic patients with COVID-19, 108 were from the FC group and 70 the NF group. Demographics, clinical characteristics, treatment outcomes, and laboratory findings on admission are summarized in Tables 1, 2. There were no significant differences in sex and age between the two groups (*p* = 0.066 and *p* = 0.105, respectively), with the mean age being 47.3 ± 16.3 years and 43.6 ± 13.4 years in the FC and NF groups, respectively. Overall, 76.4% of the patients had recently traveled to or resided in epidemic areas, and there was no significant difference (*p* = 0.363) between the two groups. Hypertension (15.7%) and diabetes mellitus (8.4%) were the most frequent comorbidities, and the most common symptoms were fever (71.9%) and cough (48.9%); these did not show significant differences between the FC and NF groups. Furthermore, no significant difference was observed in the interval from symptom onset to hospital admission between patients in the two groups (*p* = 0.714). Compared with the NF group, the FC group had a longer mean duration of exposure to infection sources (13.1 vs. 8.9 days, *p* < 0.001), viral shedding (21.5 vs. 15.9 days, *p* < 0.001), and hospital stay (39.2 vs. 22.2 days, *p* < 0.001). The number of severe or critical cases in the FC group was significantly higher than that in the FC group (*p* = 0.036). There were no significant differences in the number of deaths (*p* = 0.280), ICU admissions (*p* = 0.379), and the length of ICU stay (*p* = 0.666) between the two groups. Among the laboratory parameters tested on admission, the lymphocyte count level was significantly lower in the FC group than in the NF group (*p* = 0.044), even after stratified analysis (*p* < 0.001).

**Table 1:** Demographics and clinical characteristics of patients with COVID-19 pneumonia between FC and NF groups.

**Characteristics**	**Total** **(*n* = 178)**	**FC group (*n* = 108)**	**NF group (*n* = 70)**	***p*-value**
**Age (years), mean** **±** **SD**	45.8 ± 15.3	47.3 ± 16.3	43.6 ± 13.4	0.105
**Sex**, ***n*** **(%)**				
Male	89 (50.0)	48 (44.4)	41 (58.6)	0.066
Female	89 (50.0)	60 (55.6)	29 (41.4)	
**Exposure history within 14 days**, ***n*** **(%)**				
Recently stayed in epidemic area	136 (76.4)	80 (74.1)	56 (80.0)	0.363
Exposure to infected patients	42 (23.6)	28 (25.9)	14 (20.0)	
**Comorbidities**, ***n*** **(%)**	50 (28.1)	33 (30.6)	17 (24.3)	0.363
Hypertension	28 (15.7)	21 (19.4)	7 (10.0)	0.091
Diabetes mellitus	15 (8.4)	10 (9.3)	5 (7.1)	0.620
Coronary heart disease	5 (2.8)	4 (3.7)	1 (1.4)	0.650
Hepatitis	8 (4.5)	3 (2.8)	5 (7.1)	0.266
COPD	4 (2.2)	2 (1.9)	2 (2.9)	0.647
Cancer	4 (2.2)	3 (2.8)	1 (1.4)	1.000
**Signs and symptoms on admission**, ***n*** **(%)**				
Fever	128 (71.9)	82 (75.9)	46 (65.7)	0.139
37.3–38°C	81 (63.3)	51 (62.2)	30 (65.2)	1.000
38.1–39°C	40 (31.3)	26 (31.7)	14 (30.4)	
>39°C	7 (5.45)	5 (6.1)	2 (4.3)	
Cough	87 (48.9)	57 (52.8)	30 (42.9)	0.196
Sputum	21 (11.8)	13 (12.0)	8 (11.4)	0.902
Nasal congestion	7 (4.0)	2 (1.9)	5 (7.2)	0.114
Headache	12 (6.7)	8 (7.4)	4 (5.7)	0.767
Sorethroat	32 (18.0)	19 (17.6)	13 (18.6)	0.868
Fatigue	16 (9.0)	8 (7.4)	8 (11.4)	0.360
Myalgia	13 (7.3)	9 (8.3)	4 (5.7)	0.512
Chest pain	8 (4.5)	6 (5.6)	2 (2.9)	0.483
Diarrhea	11 (6.2)	7 (6.5)	4 (5.7)	1.000
Chills	10 (5.6)	7 (6.5)	3 (4.3)	0.742
**Disease severity**, ***n*** **(%)**				
Mild/moderate	141 (79.2)	80 (74.1)	61 (87.1)	0.036^*^
Severe/critical	37 (20.8)	28 (25.9)	9 (12.9)	
**ICU admission**, ***n*** **(%)**	17 (9.6)	12 (11.1)	5 (7.1)	0.379
**Clinical outcomes**, ***n*** **(%)**				
Discharge from hospital	175 (97.6)	105 (97.2)	70 (100.0)	0.280
Death	3 (1.7)	3 (2.8)	0 (0)	
**Length (days), mean** **±** **SD and median (IQR) ^#^**				
Exposure to infection sources	11.6 ± 6.8	13.1 ± 7.1	8.9 ± 5.3	<0.001^*^
	14.0 (6.0–14.0)	14.0 (7.0–16.0)	8.5 (3.3–14.0)	
From symptom onset to hospital admission	4.6 ± 5.1	4.8 ± 5.1	4.5 ± 5.1	0.714
	3.0 (2.0–6.0)	3.0 (1.3–6.8)	3.0 (2.0–5.0)	
Duration of viral shedding	19.3 ± 10.3	21.5 ± 10.9	15.9 ± 8.3	<0.001^*^
	17.0 (13.0–24.0)	18.0 (15.0–26.3)	14.0 (9.8–22.0)	
Hospital stay	32.4 ± 17.7	39.2 ± 17.7	22.2 ±11.9	<0.001^*^
	28.0 (19.0–43.0)	34.0 (25.5–56.5)	19.5 (14.0–30.0)	
ICU stay	23.4 ± 43.6	26.5 ± 52.2	16.0 ± 6.3	0.666
	11.0 (7.5–19.0)	10.0 (6.3–14.8)	18.0 (9.5–21.5)	

FC, familial cluster; NF, non- familial; COPD, chronic obstructive pulmonary disease; ICU, intensive care unit; IQR, interquartile range; SD, standard deviation.

The disease severity was based on the most serious degree of COVID-19 during hospitalization.

# Sample size of events.

Duration of exposure to infection sources was calculated in 168 patients (10 cases from the NF group were unavailable. FC group = 108, NF group = 60).

Duration of viral shedding was calculated in 176 patients (two deaths without virus clearance were excluded. FC group =106, NF group = 70).

Length of hospital stay was calculated in 175 discharged patients (three deaths were excluded. FC group =105, NF group = 70).

Length of ICU stay and interval time from admission to ICU admission were calculated in 17 patients (FC group =12, NF group = 5).

* *p < 0.05, comparison between FC and NF groups*.

**Table 2:** Comparison of initial laboratory findings between FC and NF groups.

**Parameters**	**Total** **(*n* = 178)**	**FC group** **(*n* = 108)**	**NF group** **(*n* = 70)**	***p*-value**
WBC count (normal range, 4.0–9.5 × 10^9^/L)	5.35 ± 2.60	5.13 ± 2.89	5.68 ± 2.05	0.163
<4	53/178 (29.8)	37/108 (34.3)	16/70 (22.9)	0.261
4–10	118/178 (66.3)	67/108 (62.0)	51/70 (72.9)	
>10	7/178 (3.9)	4/108 (3.7)	3/70 (4.3)	
Neutrophil count (normal range, 1.8–6.3 × 10^9^/L)	3.33 ± 2.26	3.22 ± 2.57	3.53 ± 1.58	0.395
>6.3	10/170 (5.9)	6/108 (5.6)	4/62 (6.5)	1.000
Lymphocyte count (normal range, 1.1–3.2 × 10^9^/L)	1.52 ± 2.05	1.20 ± 0.51	2.00 ± 3.15	0.044^*^
<1.1	74/172 (43.0)	57/104 (54.8)	17/68 (25.0)	<0.001^*^
C-reactive protein (normal range, ≤ 6 mg/L)	12.27 ± 22.68	12.74 ± 22.87	11.50 ± 22.52	0.730
>10	40/171 (23.4)	27/107 (25.2)	13/64 (20.3)	0.462
ALT (normal range, 9–50 U/L)	24.47 ± 23.63	22.26 ± 14.91	28.45 ± 33.95	0.184
>50	14/168 (8.3)	6/108 (5.6)	8/60 (13.3)	0.081
AST (normal range, 15–40 U/ L)	26.10 ± 15.27	24.81 ± 11.22	28.33 ± 20.42	0.213
>40	18/170 (10.6)	8/108 (7.4)	10/62 (16.1)	0.075
LDH (normal range, 120–250 U/L)	194.21 ± 80.91	190.78 ± 56.35	198.12 ± 102.75	0.704
>250	13/77 (16.9)	6/41 (14.6)	7/36 (19.4)	0.574
D-dimer (normal range, 0–0.5 mg/L)	0.44 ± 1.59	0.44 ± 1.93	0.43 ± 0.68	0.962
>0.5	22/170 (12.9)	12/108 (11.1)	10/62 (16.1)	0.348
Procalcitonin (normal range, 0–0.1 ng/mL)	0.20 ± 0.48	0.22 ± 0.60	0.16 ± 0.12	0.475
>0.5	6/170 (3.5)	4/108 (3.7)	2/62 (3.2)	1.000

Data were expressed as mean ± SD or n (%); FC, familial cluster; NF, non-familial; SD, standard deviation; WBC, white blood cell; ALT, alanine aminotransferase; AST, aspartate aminotransferase; LDH, lactose dehydrogenase;

* *p < 0.05, comparison between FC and NF groups*.

### Comparison of CT Images at Two Time Points

**Figure 1:**
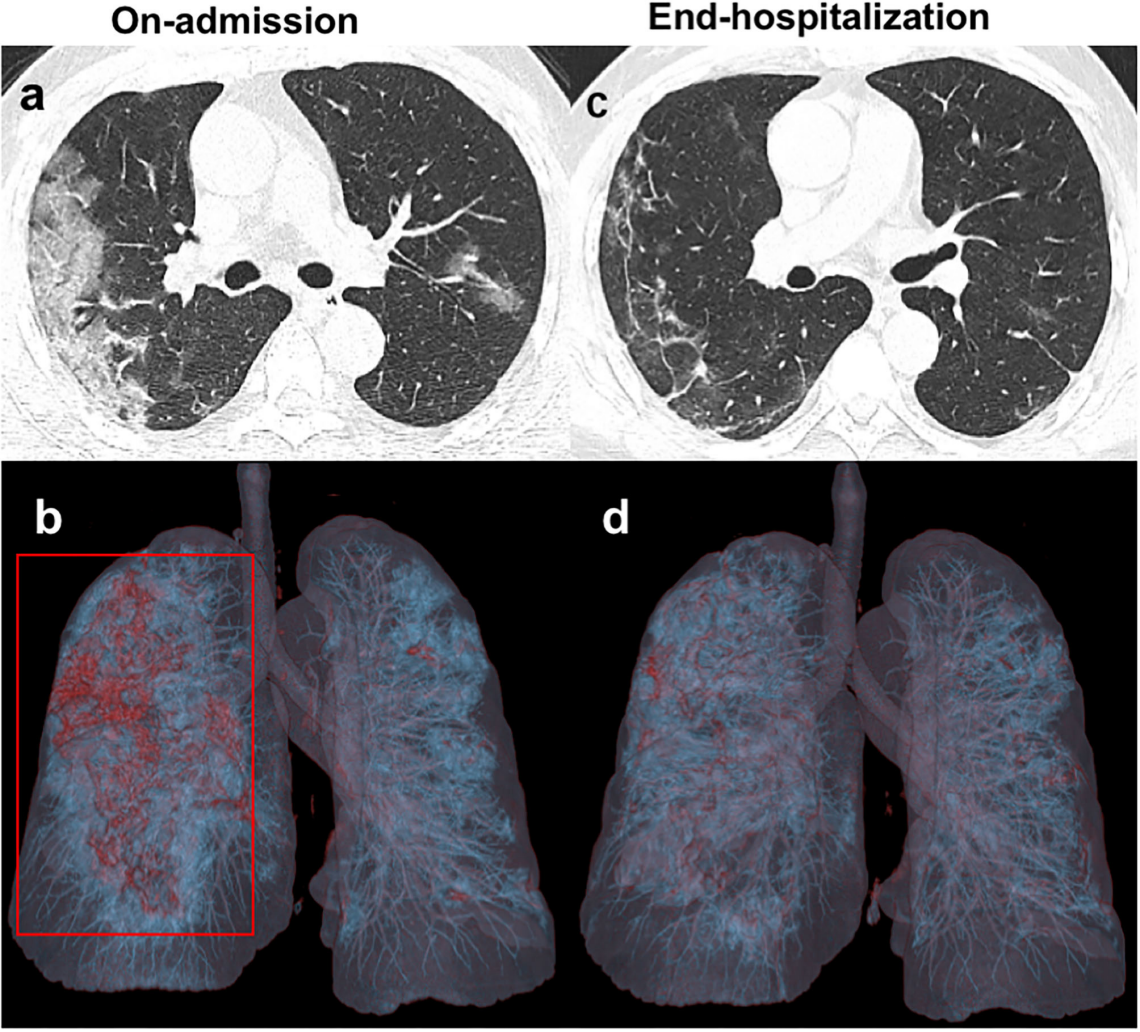
Unenhanced chest CT images of a 41 years old patient infected with COVID-19 from the NF group. **(a,b)** Axial images obtained on-admission show extensive ground-glass opacities (GGO) in the subpleural regions of both lungs. Three-dimensional volume-rendered reconstruction (3D-VR) image mainly presents as a red lesion in the right lung (red box). **(c,d)** Follow-up CT images obtained at end- hospitalization show significant absorption of GGO with irregular linear opacities.

**Figure 2:**
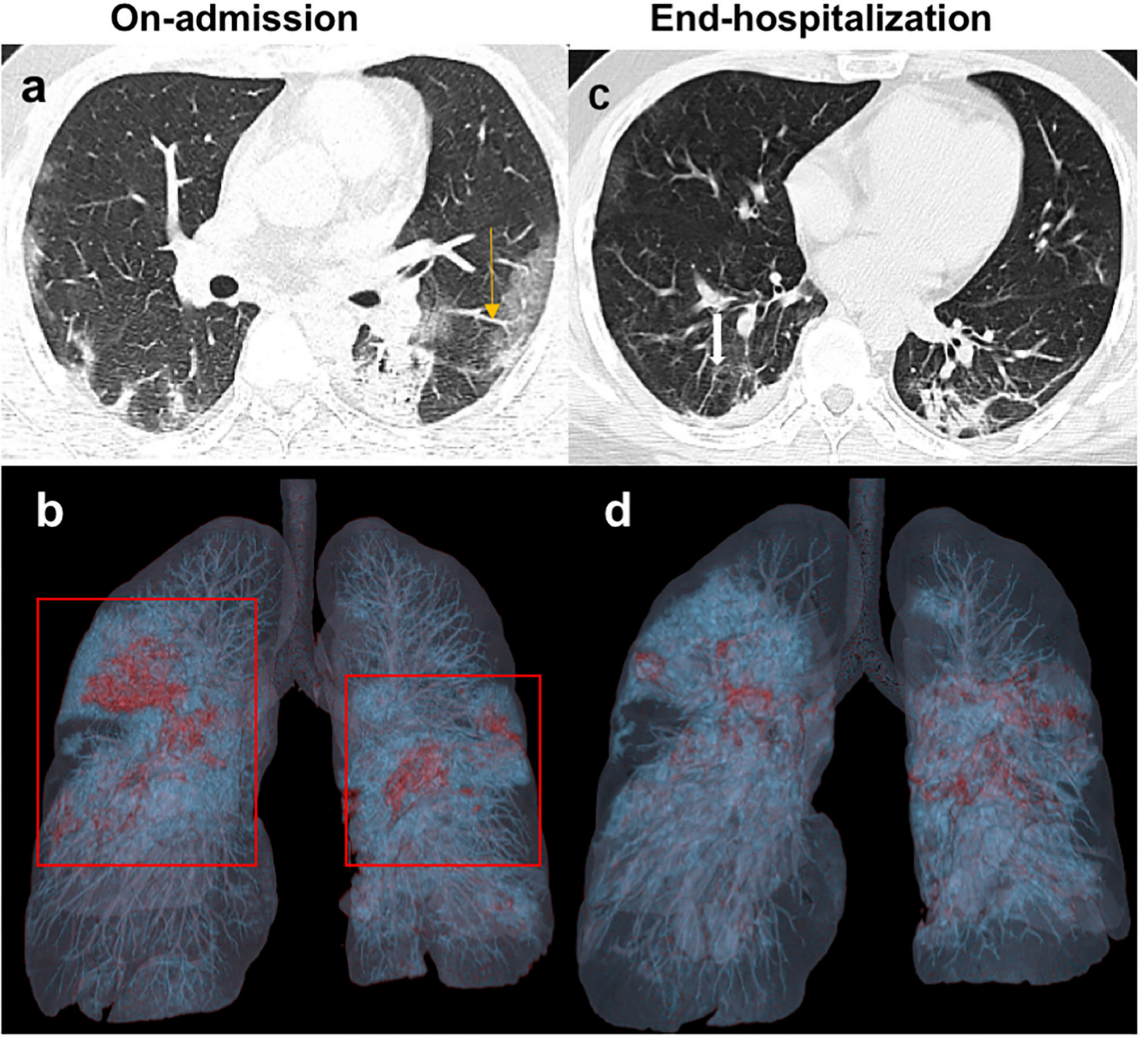
Unenhanced chest CT images of a 32-year-old man with COVID-19 infection from FC group. **(a)** Axial image on-admission shows the mixed pattern of multifocal bilateral GGO and consolidation in the subpleural regions of the lungs. The enlarged pulmonary vessels also could be seen (yellow arrow). **(b)** 3D-VR image of chest CT scan on-admission presents as red and blue dense lesions (red box). **(c)** At the end of hospitalization, follow-up CT shows the consolidation evidently resolved with residual irregular linear opacities (white arrow). **(d)** The previous red and blue dense lesion in the 3D-VR image was absorbed gradually.

The major CT features of the FC and NF groups were compared at two time points: on admission and during end-hospitalization (Table 3). On admission, the number of involved lung lobes in the FC group was significantly higher than that in the NF group (3.0 vs. 2.3, *p* = 0.017). Peripheral GGO with multiple lung lobe involvement was the most frequent CT feature (Figures 1a,b, 2a,b). In addition, pleural thickening was noted in about one-quarter of the patients, and other findings, such as enlarged pulmonary vessels (Figure 2a), air bronchogram, lymphadenopathy, and pleural effusion, were found in some patients in the two groups. Nevertheless, such CT findings showed no significant differences between the two groups. At end-hospitalization, the sum severity CT scores (4.6 vs. 2.7, *p* = 0.005) and the number of involved lung lobes (3.6 vs. 1.7, *p* < 0.001) were higher in the FC group than in the NF group, and the FC group had more involved lung lobes with abnormalities. Consolidation and mixed pattern findings were largely absorbed with peripheral linear opacities in the FC group (Figures 1c,d, 2c,d) and showed a complete resolution in 41.1% of patients in the NF group, with a significant difference between the two groups (*p* < 0.001).

**Table 3:** Comparison of CT features at different time points between FC and NF groups.

	**On-admission**				**End-hospitalization**			
	**Total** **(*n* = 147)**	**FC group** **(*n* = 87)**	**NF group** **(*n* = 60)**	***p*-value**	**Total** **(*n* = 127)**	**FC group** **(*n* = 71)**	**NF group** **(*n* = 56)**	***p*-value**
**Sum severity CT scores**	3.5 ± 3.1	3.9 ± 3.2	3.0 ± 2.9	0.059	3.8 ± 3.8	4.6 ± 3.1	2.7 ± 4.2	0.005^*^
**Predominant CT pattern**				0.226				<0.001^*^
Normal	28 (19.0)	14 (16.1)	14 (23.3)		27 (21.3)	4 (5.6)	23 (41.1)^*^	
GGO	77 (52.4)	52 (59.8)	25 (41.7)		48 (37.8)	29 (40.8)	19 (33.9)	
Consolidation	14 (9.5)	7 (8.0)	7 (11.7)		9 (7.1)	7 (9.9)	2 (3.6)	
Mixed pattern	22 (15.0)	12 (13.8)	10 (16.7)		7 (5.5)	4 (5.6)	3 (5.4)	
Linear opacities	6 (4.1)	2 (2.3)	4 (6.7)		36 (28.3)	27 (38.0)	9 (16.1)^*^	
**Number of involved lobes**	2.7 ± 1.9	3.0 ± 1.9	2.3 ± 1.9	0.017^*^	2.8 ± 2.0	3.6 ± 1.6	1.7 ± 1.9	<0.001^*^
				0.156				<0.001^*^
0	28 (19.0)	14 (16.1)	14 (23.3)		27 (21.3)	4 (5.6)	23 (41.1)^*^	
1	22 (15.0)	9 (10.3)	13 (21.7)		17 (13.4)	6 (8.5)	11 (19.6)	
2	19 (12.9)	11 (12.6)	8 (13.3)		13 (10.2)	7 (9.9)	6 (10.7)	
3	16 (10.9)	9 (10.3)	7 (11.7)		12 (9.4)	9 (12.7)	3 (5.4)	
4	21 (14.3)	16 (18.4)	5 (8.3)		16 (12.6)	13 (18.3)	3 (5.4)	
5	41 (27.9)	28 (32.2)	13 (21.7)		42 (33.1)	32 (45.1)	10 (17.9)^*^	
**Other CT findings**								
Pleural effusion	5 (3.4)	4 (4.6)	1 (1.7)	0.649	5 (3.9)	4 (5.6)	1 (1.8)	0.383
Lymphadenopathy	11 (7.5)	9 (10.3)	2 (3.3)	0.200	8 (6.3)	7 (9.9)	1 (1.8)	0.077
Air bronchogram	27 (18.4)	17 (19.5)	10 (16.7)	0.658	0 (0)	0 (0)	0 (0)	/
Enlarged vessels	30 (20.4)	14 (16.1)	16 (26.7)	0.118	5 (3.9)	4 (5.6)	1 (1.8)	0.383
Pleural thickening	38 (25.9)	21 (24.1)	17 (28.3)	0.568	25 (19.7)	16 (22.5)	9 (16.1)	0.363
**Distribution of lesions**				0.222				<0.001^*^
Peripheral	96 (65.3)	57 (65.5)	39 (65.0)		71 (55.9)	47 (66.2)	24 (42.9)^*^	
Random	18 (12.2)	11 (12.6)	7 (11.7)		14 (11.0)	11 (15.5)	3 (5.4)	
Diffuse	5 (3.4)	5 (5.7)	0 (0)		15 (11.8)	9 (12.7)	6 (10.7)	
Normal	28 (19.0)	14 (16.1)	14 (23.3)		27 (21.3)	4 (5.6)	23 (41.1)^*^	

Data were expressed as mean ± SD or n (%); FC, familial cluster; NF, non-familial; GGO, ground-glass opacities; SD, standard deviation.

* *p < 0.05, comparison between FC and NF groups*.

### Comparison of Quantitative CT Changes Between the FC and NF Groups

The differences in the sum severity CT scores and the number of involved lung lobes between admission and end-hospitalization (Δsum and Δnumber, respectively), were analyzed to evaluate the changes in chest CT findings and compared between the two groups (Table 4). Compared to the NF group, the Δsum (*p* = 0.168) and Δnumber (*p* < 0.001) were lower in the FC group.

**Table 4:** Comparison of Δsum CT scores and Δnumber of involved lung lobes between FC and NF groups.

	**Total** **(*n* = 111)**	**FC** **(*n* = 62)**	**NF** ***(n* = 49)**	***p*-value**
Δ sum CT scores (OA-EH)	0.2 ± 2.8	−0.1 ± 3.1	0.6 ± 2.4	0.168
Δ number of involved lobes (OA-EH)	0.1 ±1.4	−0.3 ± 1.4	0.6 ± 1.2	<0.001^*^

FC, familial cluster; NF, non-familial; Δ, differences between two time points; OA, on-admission; EH, end-hospitalization;

* *p < 0.05, comparison between FC and NF groups*.

## Discussion

To the best of our knowledge, this is the first retrospective cohort study comparing the differences between FC and NF groups of patients with COVID-19 pneumonia. In this study, we found that the severity of this disease differed between the two groups. The results showed that FC patients had a longer and more adverse clinical course. The FC group had a higher proportion of patients with severe or critical illnesses than the NF group. More patients in the FC group had a decreased lymphocytes count level. CT evaluation indicated that the FC group showed a higher severity of lung abnormalities, whereas the NF group showed an improvement in the severity of lung opacities.

In this study, compared with the NF group, the FC group had a longer clinical course, including a longer duration of viral shedding and hospital stay, and had more cases of severe or critical illness. This finding may imply that patients in the FC group were more severely affected by COVID-19 pneumonia than patients in the NF group. In general, the median duration of virus shedding in our study was 17.0 days. The first study from Wuhan examining virus shedding durations in case of detectable SARS-CoV-2 RNA reported a median time of 20 days (16), and a later study, also from Wuhan, showed the median time to be 25.0 days (20). Our result is consistent with the results of other studies outside Wuhan that reported a median duration of 17 days (17, 21), implying that the viral clearance period outside Wuhan might be relatively shorter. In our study, the FC group tended to have a longer virus-shedding period and more cases of severe or critical illness; hence, patients from the FC group had a prolonged hospital stay. We assumed that such a difference in outcome between the two groups might be caused by the difference in duration of exposure to infection since our results showed that the FC group had a longer exposure time than the NF group. This is because patients from the FC group had been in close contact with other infected family members multiple times during the day, whereas contact with infected individuals was occasional for NF patients, with them being exposed only to infection sources. In addition, due to co-habitation in limited and poorly ventilated rooms, the chances for high viral concentrations in the air increased. Consequently, with an increase in the duration of exposure and virus concentration, the dose of SARS-CoV-2 exposure in the FC group would be larger than that in the NF group. Furthermore, the exposure dose was identified to be associated with the viral load as well as the severity of COVID-19 (22). Several studies revealed that individuals with a high viral load and a long period of virus-shedding had poor clinical outcomes (23, 24), similar to the observations in cases of SARS (25). In general, further studies are necessary to elucidate the difference in viral dose between the two groups. With regard to the laboratory tests, more patients in the FC group than in the NF group had lower lymphocyte count levels, which might be one of the causes of the poorer outcomes. Several prior studies had confirmed that lymphocyte count level was an independent factor associated with the severity of the disease and duration of SARS-CoV-2 RNA clearance (20, 21, 26, 27). It has been established that host factors could strike an immune response to the anti-virus in the process of virus infection. However, the virus might directly attack the lymphocytes, and the subsequent lymphopenia is related to induced T-cell apoptosis and cellular immune depletion (28, 29). Together, these findings suggest that the immune ability and the capacity to clear the virus were reduced in the FC group. Although the FC group had a longer length of ICU stay (26.5 days) than the NF group (16.0 days), a significant difference between both groups in the mean duration of ICU stay was not observed in this study. Two patients who died and had a shorter stay in the ICU (time from ICU admission to death: 14 days and 15 days) were included in the analysis, which might have shortened the average duration of ICU stay in the FC group.

On admission, we noted that the majority of patients in both groups were presented with multiple lung lobes involvement on chest CT, which was in accordance with some previous reports (19, 30–32). In particular, the number of involved lung lobes was significantly higher in the FC group than in the NF group. In other words, patients in the FC group were more likely to have extensive pulmonary involvement. We believed that the difference in the lung abnormalities between the two groups was probably due to the difference in lymphocyte count. Multiple lesions were found in multiple lobes of both lungs in this disease, unlikely typically observed in bacterial pneumonia (33). Lymphocytes count, one of the hallmarks of virus infection, plays a significant antiviral role in the maintenance of homeostasis and inflammatory response throughout the body by managing the fight against pathogens. The decreasing lymphocyte count indicates immune insufficiency or misdirection, which may increase viral replication and cause tissue damage (28). Wu et al. showed that there was a significant negative correlation between the degree of pulmonary inflammation on chest CT and the lymphocyte count (34). Another previous study from Tordjman et al. reported moderate correlations between the severity of CT findings and the lymphocyte count in COVID-19 (35). In our study, more patients in the FC group had a decreased lymphocyte count level, leading to worse CT findings.

At end-hospitalization, the sum severity CT scores and the number of involved lung lobes were significantly higher in the patients in the FC group than in those in the NF group. Accordingly, the NF group presented a higher Δnumber and fewer involved lung lobes at end-hospitalization after regular treatment. We inferred that the primary cause of the differences in CT evolution and outcomes was a result of the differences in virus shedding periods between the two groups. As our study demonstrated that the FC group had a longer viral shedding period, the lung abnormalities resolved more slowly in that group than in the NF group. The result is consistent with two previous reports. Xu et al. stated that patients with late SARS-CoV-2 RNA clearance had a slower focal resolution on radiograph images than patients with shorter virus shedding duration (17). Another study among 140 healthcare workers showed that the duration from illness onset to improvement in chest CT findings was conspicuously related to the viral shedding duration of SARS-CoV-2 (20). However, the association between prolonged SARS-CoV-2 shedding and delayed recovery (according to radiograph findings) as well as the underlying pathological process requires further study.

Our findings suggest that familial infection may be an important risk factor of adverse COVID-19 prognosis; thus, for COVID-19 patients in familial clusters that can be identified, close monitoring and timely treatment are necessary to improve prognosis. After hospital discharge, further isolation and follow-up may be needed for these patients considering their longer virus shedding periods and more residual lung abnormalities.

The study has several limitations. First, the quantitative method of measuring the severity of CT scores may involve certain subjectivity. Second, due to the exclusion of children and patients asymptomatic on admission, the findings of this study can only be applied to symptomatic adult patients with SARS-CoV-2 infection. Third, only patient data from Guangdong Province were reviewed.

## Conclusion

In conclusion, this study demonstrated that the FC group had poorer clinical outcomes than did the NF group. The prolonged virus shedding period, longer hospital stay, and slower resolution of lung abnormalities on chest CT were associated with the severity of the disease in the FC group patients. Our findings suggest that the lower lymphocyte count level might contribute to the adverse outcomes in the FC group. In this study, the severity of SARS-CoV-2 infection differed between the FC and NF groups during hospitalization. Therefore, close monitoring during treatment and follow-up after discharge could be beneficial for patients who are part of familial clusters.

## Data Availability Statement

The original contributions presented in the study are included in the article/supplementary material, further inquiries can be directed to the corresponding author/s.

## Ethics Statement

The studies involving human participants were reviewed and approved by The First Affiliated Hospital of Jinan University. Written informed consent for participation was not required for this study in accordance with the national legislation and the institutional requirements.

## Author Contributions

SZ, YZ, SL, and HY: conception and design. SL, WL, BZ, and JY: provision of study materials or patients. WL, JL, LZ, QZ, and LC: collection and assembly of data. BZ, SL, and HY: data analysis and interpretation. All authors: manuscript writing and final approval of manuscript.

## Conflict of Interest

The authors declare that the research was conducted in the absence of any commercial or financial relationships that could be construed as a potential conflict of interest.
